# The sex ratio of singleton and twin delivery offspring in assisted reproductive technology in China

**DOI:** 10.1038/s41598-017-06152-9

**Published:** 2017-08-24

**Authors:** Mengxi Chen, Jiangbo Du, Jing Zhao, Hong Lv, Yifeng Wang, XiaoJiao Chen, Junqiang Zhang, Lingmin Hu, Guangfu Jin, Hongbing Shen, Zhibin Hu, Fang Xiong, Li Chen, Xiufeng Ling

**Affiliations:** 1 State Key Laboratory of Reproductive Medicine, Nanjing Medical University, Nanjing, 211166 China; 2 Department of Epidemiology, School of Public Health, Nanjing Medical University, Nanjing, 211166 China; 3 Department of Reproduction, the Affiliated Nanjing Maternity and Child Health Hospital of Nanjing Medical University, Nanjing, 210004 China; 4 Department of Reproduction, the Affiliated Changzhou Maternity and Child Health Hospital of Nanjing Medical University, Changzhou, 213003 China; 5 Department of Reproduction, the Affiliated Wuxi Maternity and Child Health Hospital of Nanjing Medical University, Wuxi, 214002 China

## Abstract

In order to evaluate the impact of assisted reproductive technology (ART) procedure and individual factors on the sex ratio of singletons and twins at birth after *in vitro* fertilization (IVF) and intra-cytoplasmic sperm injection (ICSI) treatment in China. We conducted a retrospective cohort study including patients undergoing their first cycle of IVF or ICSI with autologous oocytes from 2001 to 2015. A total of 7410 babies were born from 5832 women with 7410 live birth. The secondary sex ratio (SSR) in singletons was significantly higher than twins (53.97% vs. 50.89%, *P* = 0.009). The largest disparity was observed in ‘thawed blastocyst embryos ICSI’ subgroup that SSR was 59.84% in singletons and 42.45% in twins (*P* = 0.013). Blastocyst transfer was positively associated with elevated SSR when compared to cleavage stage embryos in singletons (Odds Ratio [OR] = 1.17, *P* < 0.001). In addition, paternal age was significantly associated with SSR (OR = 0.75, *P* = 0.014). While the decrease of SSR was significantly associated with ICSI when compared to IVF (OR = 0.61, *P* = 0.046) in twins. Blastocyst transfer increases SSR in comparing with cleavage stage embryos in singletons, while the use of ICSI reduces SSR in twins. Our findings offered important complement for better understanding the underlying determinant of SSR in ART offspring.

## Introduction

Primary sex ratio (PSR) means the sex ratio at conception and is always calculated as male proportion. It is 1.7 times higher than female under completely natural circumstances in humans^1^. While the sex ratio at birth, which also called secondary sex ratio, shows dramatic decrease due to the higher spontaneous abortion or other sex-selective loss during pregnancy^2^. So the SSR in most gender-neutral countries is around 1.05, which is an ideal level for keeping a minimized overall gender imbalance. Recent evidence further clarified that the trajectory of sex ratio disproportion from conception to birth is dynamic and influenced by multiple complex factors both endogenous and exogenous^3^. Numerous biological and environmental factors have been shown to reduce the secondary sex ratio, including older maternal and paternal ages^4^, stressors (i.e. earthquakes^5^, war^6^ and economic distress)^7^, and toxins (i.e. smoking, pollutants, and pesticides)^8^. What’s more, social factors for male sex preference and sex selection in certain countries rise the SSR in natural conception^9^.

Over the past decades, with the rapid popularity of assisted reproductive technology (ART) for infertility treatment, a growing number of babies were born from IVF or ICSI^10^. It was estimated that ART has contributed to the birth of over 5 million live born babies worldwide^11^. Notably, as women undergoing ART received Ovulation induction treatment and the embryos cultured *in vitro* for some time, the key to sex ratio in IVF and/or ICSI babies were not completely similar with natural conception. Studies based on preimplantation genetic diagnosis (PGD) have revealed that the PSR of IVF embryos were significantly higher than ICSI embryos^12–14^. While several large population survey using ART databases indicated that the SSR of cleavage-stage embryos transfer is remarkable lower when compared with those of blastocyst-stage^15, 16^. Besides, a recent research reported that male with higher BMI during ART procedure has an increased probability of giving birth to male singletons^17^.

Although cumulating studies support the view that ART population exhibited skewed gender distribution compared with natural conception population, no clear conclusions have been made. Additionally, to maximize the success rate, more than one embryo was transferred during the ART treatment, which greatly increased the incidence of twins or multi-fetus^18^. However, the sex ratio at birth for multiple delivery has not been discussed due to its complex situations. Thus, twin delivery occupied a large proportion in ART birth, and the sex ratio of twins deserved in-depth analysis. What’s more, there’s no study discuss the SSR of ART offspring between singletons and twins. Therefore, we conducted a retrospective cohort study including 7410 ART offspring to comprehensively investigate the potential determinants of SSR among both the singletons and twins.

## Results

### Characteristics of the patients

The study population was from three reproductive medicine centers in Jiangsu province (Nanjing, Wuxi and Changzhou) of China. The mean maternal and paternal age was 29.87 and 31.89, respectively. Primary infertility is more than secondary infertility with a ratio of 53.8% and the major cause of infertility was tubal factor (40.5%). In these three centers, long-term protocol (66.1%) was the main therapy and the average dose of gonadotropin (Gn) was 1597.23 IU for an average course of 8.02 days (Supplementary Table 1). In total, there were 7410 babies born to 5832 women. There were 3902 male babies and 3508 female babies with a SSR of 52.66% (Table 1).

**Table 1:** Basic characteristics of the patients undergoing ART treatment stratified by singletons and twins. ^a^n(%); ^b^mean ± SD.

Selected characteristics	Singletons	Twins	*P*
No. of live birth^a^	4254 (57.4)	3156 (42.6)	
Maternal age (years)^b^	30.0 ± 4.0	29.5 ± 3.7	<0.001
Paternal age (years)^b^	32.1 ± 5.0	31.4 ± 4.9	<0.001
Maternal BMI (kg/m^2^)^b^	22.0 ± 3.0	21.9 ± 2.9	0.383
Paternal BMI (kg/m^2^)^b^	24.3 ± 3.1	24.1 ± 3.1	0.189
Duration of infertility^b^	3.0 ± 2.9	3.1 ± 2.7	0.397
Infertility type^a^			0.069
Primary	2169 (53.0)	846 (55.8)	
Secondary	1920 (47.0)	671 (44.2)	
Cause of infertility^a^			0.343
Male factor	337 (11.0)	149 (12.6)	
Female factor	1868 (61.0)	707 (59.7)	
Mixed (Both male and female factors)	857 (28.0)	329 (27.8)	

### SSR stratified by different characteristics

The sex ratio was stratified by patient’s main demographic characters, the category of infertility, different ART procedures, and singletons or twins (Table 2). A significant higher SSR was observed in offspring of parents who underwent thawed embryos transfer compared with fresh embryos transfer (SSR: 54.16% vs. 51.04%, *P* = 0.008). In addition, the SSR was significantly higher towards males in blastocyst transfer compared with those cleavage stage embryos transfer (SSR: 57.89% vs. 51.12%, *P* < 0.001). There was a significantly higher male proportion for singletons when compared with twins (SSR: 53.97% vs. 50.89%, *P* = 0.009).

**Table 2:** Sex ratio of live birth offspring stratified by different demographic characteristics, ART procedures and type of birth.

Selected characteristics	Total	Male	Female	SSR(%)	*P*
All	7410	3902	3508	52.66	
Groups of maternal age					0.426
<30	4367	2320 (59.8)	2047 (58.8)	53.13	
≥30	2993	1561 (40.2)	1432 (41.2)	52.16	
Maternal BMI					0.913
<18.5	725	386 (10.0)	339 (9.8)	53.24	
18.5–25	5475	2879 (75.0)	2596 (75.0)	52.58	
≥25	1101	575 (15.0)	526 (15.2)	52.23	
Groups of paternal age					0.145
<32	3862	2072 (53.8)	1790 (52.1)	53.65	
≥32	3423	1777 (46.2)	1646 (47.9)	51.91	
Paternal BMI					0.502
<18.5	119	57 (1.8)	62 (2.2)	47.90	
18.5–24.9	3770	2011 (62.6)	1759 (62.1)	53.34	
≥25	2155	1144 (35.6)	1011 (35.7)	53.09	
Infertility type					0.817
Primary	3861	2044 (54.4)	1817 (54.1)	52.94	
Secondary	3262	1717 (45.6)	1545 (45.9)	52.64	
Cause of infertility					0.361
Mixed (Both male and female factors)	1515	784 (27.2)	731 (28.7)	51.75	
Male	635	349 (12.1)	286 (11.2)	54.96	
Female	3280	1748 (60.7)	1532 (60.1)	53.29	
Ovulation-inducing treatments					0.146
Long-term protocol	4332	2309 (67.7)	2023 (66.0)	53.30	
Other protocols	2146	1102 (32.3)	1044 (34.0)	51.35	
Types of embryos transferred					0.008
Fresh	3562	1818 (46.6)	1744 (49.7)	51.04	
Thawed	3848	2084 (53.4)	1764 (50.3)	54.16	
Fertilization methods					0.100
IVF	5381	2861 (83.6)	2520 (82.1)	53.17	
ICSI	1111	560 (16.4)	551 (17.9)	50.41	
Stage of embryo transferred					<0.001
Cleavage-stage embryo	5661	2894 (77.2)	2767 (81.6)	51.12	
Blastocyst	1477	855 (22.8)	622 (18.4)	57.89	
Type of birth					0.009
Singletons	4254	2296 (58.8)	1958 (55.8)	53.97	
Twins	3156	1606 (41.2)	1550 (44.2)	50.89	
Location of fertility center					0.221
Nanjing	4608	2455 (62.9)	2153 (61.4)	53.28	
Wuxi	1222	617 (15.8)	605 (17.2)	50.49	
Changzhou	1580	830 (21.3)	750 (21.4)	52.53	

SSR: secondary sex ratio, the sex ratio at birth; IVF: *In vitro* fertilization; ICSI: Intra-cytoplasmic sperm injection.

### Influencing factors of SSR in singletons

For singletons, we observed that the blastocyst transfer was still significantly associated with higher SSR in multivariable logistic regression analysis (OR = 1.17, 95%CI: 1.08–1.27, *P* < 0.001) after adjusting for maternal and paternal age, cause of infertility. What’s more, the paternal age was also significantly associated with a decrease of sex ratio. It skewed (OR = 0.75, 95%CI: 0.60–0.94, *P* = 0.014) in favor of females compared to those under 32 years old after adjusting for maternal age and cause of infertility (Table 3).

**Table 3:** Association between the proportion of male and different categories of selected variables for singleton pregnancy.

Selected characteristics	n (%)		OR (95% CI)	*P* ^a^
	Males	Females		
Total	2297 (54.0)	1957 (46.0)		
Groups of maternal age				
<30	1311 (57.4)	1068 (54.9)	1.00	
≥30	972 (42.6)	877 (45.1)	0.94 (0.74–1.20)	0.623
Maternal BMI				
<18.5	238 (10.5)	173 (9.0)	1.18 (0.92–1.51)	0.188
18.5–25.0	1689 (74.6)	1456 (75.4)	1.00	
≥25	338 (14.9)	301 (15.6)	0.90 (0.74–1.10)	0.290
Groups of paternal age				
<32	1196 (52.7)	928 (48.5)	1.00	
≥32	1073 (47.3)	986 (51.5)	0.75 (0.60–0.94)	0.014
Paternal BMI				
<18.5	35 (1.9)	30 (1.9)	0.97 (0.57–1.65)	0.903
18.5–25.0	1137 (61.0)	959 (62.2)	1.00	
≥25	691 (37.1)	552 (35.8)	1.03 (0.89–1.20)	0.684
Infertility type				
Primary	1177 (53.4)	992 (52.6)	1.00	
Secondary	1027 (46.6)	893 (47.4)	0.99 (0.86–1.16)	0.951
Cause of infertility				
Mixed (Both male and female factors)	446 (26.6)	411 (29.7)	1.00	
Male	204 (12.2)	133 (9.6)	1.37 (1.06–1.78)	0.016
Female	1029 (61.3)	839 (60.7)	1.12 (0.95–1.32)	0.183
Ovulation-inducing treatments				
Long-term protocols	1312 (66.3)	1058 (63.2)	1.00	
Other protocol	667 (33.7)	617 (36.8)	0.87 (0.74–1.01)	0.072
Types of embryos transferred				
Fresh	1008 (43.9)	916 (46.8)	1.00	
Thawed	1289 (56.1)	1041 (53.2)	1.15 (1.00–1.33)	0.056
Fertilization methods				
IVF	1652 (83.1)	1387 (82.7)	1.00	
ICSI	336 (16.9)	291 (17.3)	0.89 (0.71–1.11)	0.307
Stage of embryo transferred				
Cleavage-stage embryo	1673 (75.3)	1536 (80.8)	1.00	
Blastocyst	550 (24.7)	365 (19.2)	1.17 (1.08–1.27)	<0.001

^a^ *P* value of logistic regression with adjustment for maternal age, paternal age and cause of infertility.

### Influencing factors of SSR in twins

For twin pregnancy, we only included the cases of twin boys and twin girls into multivariable logistic regression, considering the sex ratio of twins were mainly determined by the ratio of twin boys and twin girls. We regarded a pair of twins as a whole and found a significant association between ICSI and IVF with a sex ratio skewed to females (OR = 0.61, 95%CI: 0.37–0.99, *P* = 0.046) (Table 4). When considering the stage of embryo transferred, we found that blastocyst transfer increases SSR in singletons, although the effect was marginal significant (OR = 1.19, 95%CI: 0.98–1.44, *P* = 0.083) in twins, the trend was in accordance with that observe in singleton pregnancy.

**Table 4:** Association between the sex proportion and different categories of selected variables for twin pregnancy.

Selected characteristics	n (%)			OR (95% CI)^a^	*P* ^b^
	Males	Females	Mixed sex		
Total	397 (15.2)	368 (23.3)	813 (51.5)		
Groups of maternal age					
<30	245 (62.0)	229 (63.4)	520 (64.2)	1.00	
≥30	150 (38.0)	132 (36.6)	290 (35.8)	1.17 (0.67–2.04)	0.576
Maternal BMI					
<18.5	34 (8.7)	43 (11.8)	80 (10.0)	0.78 (0.44–1.37)	0.380
18.5–25.0	295 (75.8)	267 (73.2)	603 (75.5)	1.00	
≥25	60 (15.4)	55 (15.1)	116 (14.5)	0.85 (0.53–1.35)	0.492
Groups of paternal age					
<32	206 (52.7)	197 (54.7)	466 (58.2)	1.00	
≥32	185 (47.3)	163 (45.3)	334 (41.8)	1.25 (0.74–2.11)	0.401
Paternal BMI					
<18.5	4 (1.2)	9 (2.9)	14 (2.1)	0.26 (0.05–1.25)	0.093
18.5–25.0	227 (67.0)	188 (61.0)	422 (62.7)	1.00	
≥25	108 (31.9)	111 (36.0)	237 (35.2)	0.84 (0.59–1.20)	0.342
Infertility type					
Primary	212 (54.8)	189 (54.8)	445 (56.7)	1.00	
Secondary	175 (45.2)	156 (45.2)	340 (43.3)	1.01 (0.71–1.43)	0.957
Cause of infertility					
Mixed (Both male and female factors)	89 (29.9)	81 (29.1)	159 (26.2)	1.00	
Male	30 (10.1)	34 (12.2)	85 (14.0)	0.81 (0.45–1.44)	0.470
Female	179 (60.1)	163 (58.6)	364 (59.9)	1.04 (0.72–1.51)	0.824
Ovulation-inducing treatments					
Long-term protocol	238 (67.6)	220 (66.7)	523 (71.6)	1.00	
Other protocols	114 (32.4)	110 (33.3)	207 (28.4)	0.93 (0.65–1.33)	0.683
Types of embryos transferred					
Fresh	189 (47.6)	197 (53.5)	433 (53.3)	1.00	
Thawed	208 (397)	171 (46.5)	380 (46.7)	1.15 (0.83–1.61)	0.408
Fertilization methods					
IVF	298 (84.4)	259 (78.2)	614 (84.2)	1.00	
ICSI	55 (15.6)	72 (21.8)	115 (15.8)	0.61 (0.37–0.99)	0.046
Stage of embryo transferred					
Cleavage-stage embryo	291 (76.6)	295 (82.4)	640 (83.2)	1.00	
Blastocyst	89 (23.4)	63 (17.6)	129 (16.8)	1.19 (0.98–1.44)	0.083

^a^The odds ratio of male twin pregnancy compared to the female twin pregnancy. ^b^ *P* value of logistic regression with adjustment for maternal age, paternal age and cause of infertility.

### Different influencing factors between singleton and twin pregnancy in ART procedure

**Figure 1:**
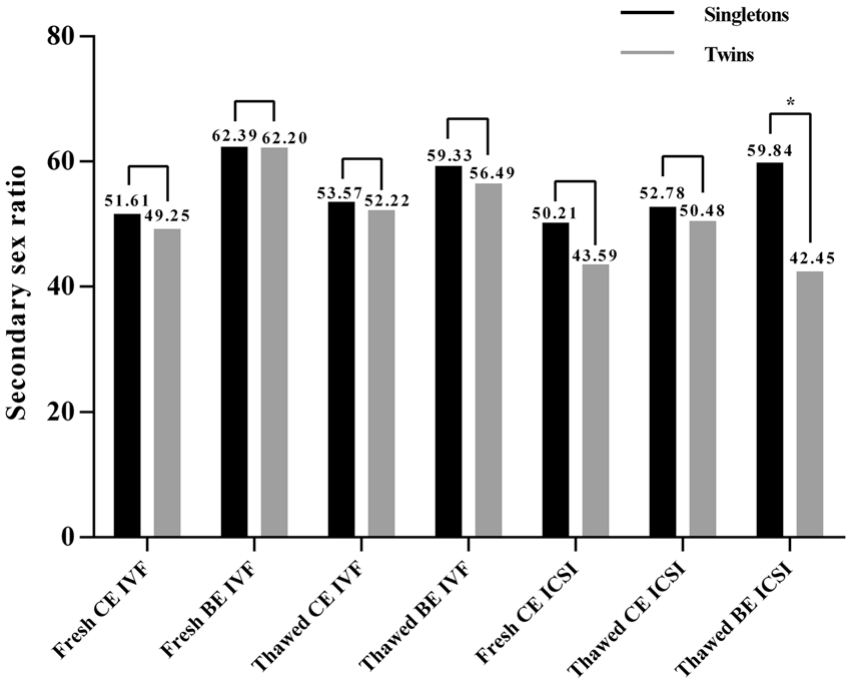
Types of birth (Singletons/Twins) on SSR. SSR = secondary sex ratio; IVF = *in vitro* fertilization; ICSI = intra-cytoplasmic sperm injection; CE = cleavage stage embryo; BE = blastocyst embryo; *P = 0.013. There’s no patient in group “Fresh BE ICSI”.

Our further analysis was grouped by ART procedure between singletons and twins. It indicated that the most obvious disparity was observed in “thawed BE ICSI” subgroup (SSR: 59.84% vs. 42.45%, *P* = 0.013) (Fig. 1). While, the most similar SSR was in “Fresh BE IVF” subgroup (62.39% vs. 62.20%).

## Discussion

Here, we conducted a retrospective study to explore the factors associated with SSR in singletons and twins born to IVF/ICSI. We found that blastocyst transfer can increase SSR both singletons and twins, and our findings firstly indicated that use of ICSI increased birth of twin girls. What’s more, paternal age and cause of infertility status may also important for the sex ratio of ART offspring.

For singleton pregnancy, recent evidences from meta-analysis and large population based studies have suggested that blastocyst embryos transfer is associated with sex ratio imbalance towards males^19, 20^ and the reasons were fully discussed. Firstly, more male embryos may be selected for embryos transfer because male embryos grow faster than female embryos in IVF or ICSI. Blastocyst embryos cultured *in vitro* for a long time as compared with cleavage-stage embryos^21, 22^. Therefore, male embryos may be easier to achieve the blastocyst embryos transfer. What’s more, higher embryo mortality in females at early post-implantation stages induced in part by abnormal inactivation in one of the two X chromosomes and the inactivation is associated with cultured *in vitro* for a long time. It is important for the sex ratio imbalance towards males^23^. In addition, blastocyst transfer has higher percentage of biochemical pregnancy losses per embryo transfer (14.1%) than cleavage-stage embryos transfer (8.2%) and it may be a reason for loss of girls^24, 25^. In our study, the SSR of blastocyst transfer is higher than cleavage-stage embryos transfer either in singletons and twins, which validated the findings by other researches. In addition, for the phenomenon that SSR is higher in thawed blastocyst transfer than that in fresh cycle transfer, the most plausible explanation is that blastocyst embryos are more likely to be frozen up and transfer later in our centers, so a large proportion of thawed embryos were blastocyst and resulted in a higher SSR.

In terms of twin pregnancy, few studies have discussed the sex ratio and relevant influencing factors due to its complex sex constitute. A retrospective study in China found that multiple delivery has no impact on sex ratio^26^. However, our findings indicated that women undergoing ICSI have trend to give birth to twin girls, which predominantly influenced the SSR of twins. We couldn’t find more powerful evidence to support our findings. For one thing, as ICSI has been mainly used to treat male factor infertility, sperm selection for ICSI may impact the SSR, as a prospective randomized study demonstrated that a statistically significant higher incidence of XX embryos derived from intra-cytoplasmic morphologically selected sperm injection was observed compared with all embryos (66.9% vs. 52.5%, *P* = 0.03), and they suggested that morphologically normal spermatozoa tend to carry the X chromosome. Therefore, X-bearing sperm may be more likely to be selected for ICSI^27^. On the other hand, several studies have proved that oocytes may receive Y-bearing spermatozoa more frequently for fertilization in IVF than that in ICSI (60% vs. 52%)^12, 23^. In addition, similar to spontaneous pregnancy, boys are more likely to be premature delivered and miscarriage, so twin girls are more likely to be born after ICSI^28, 29^. Therefore, the factors mentioned above may jointly lead to the sex ratio imbalance towards female in twin pregnancy with ICSI.

In our research, paternal age was also implicated with SSR. Those paternal age older than 32 has an imbalance towards lower SSR, which is consistent with spontaneous pregnancy. Previous evidences have indicated that older paternal age is associated with decreased blastocyst formation rate^30^. Our finding also agrees with the previous view that men over 32 years old has a rate of blastocyst for 19.5%, while there were 23.2% in others. Therefore, the phenomenon that blastocyst transfer brings about increased SSR may explain the decrease of SSR in older population.

This study also has some limitations. Firstly, we only get samples from three reproductive centers, further expansion of samples in future analysis is needed to validate our current findings. What’s more, we didn’t gain information about monozygotic twins and couldn’t distinguish them from dizygotic twins, even though we know that most of the twins are dizygotic twins, because the rate for monozygotic twins naturally is 0.4% and it is 0.9% in ART^18, 31^. So the effect of monozygotic twins is very little. Thirdly, though male infertility was reported to be a factor for increased SSR in singleton pregnancy, we couldn’t get enough information to explain it, maybe more pathological material and large sample research were needed.

In conclusion, we conducted a detailed analysis about secondary sex ratio in singletons and twins respectively. We firstly reported that compared with IVF, the use of ICSI reduces SSR in twins, and replicated that blastocyst transfer increases SSR in comparing with cleavage stage embryos transfer. Our findings were important complement for better understanding the underlying influencing factors of the sex ratio in ART offspring. Further studies with more sufficient samples and information may facilitate to confirm our findings.

## Materials and Methods

### Ethics statement

All methods and information collection protocols were approved by Nanjing Medical University and were carried out in accordance with the approved guidelines. Our research has gotten the waiver from institutional review board for the medical record review for selective variable analysis in the Nanjing Maternity and Child Health Hospital of Nanjing Medical University, the Wuxi Maternity and Child Health Hospital of Nanjing Medical University, and the Changzhou Maternity and Child Health Hospital of Nanjing Medical University.

### Study population

The ART offspring included in this study was recruited from three reproductive medicine centers of Jiangsu province in China (Nanjing, Wuxi and Changzhou) from 2001 to 2015. All the patients had undergone their first cycle of routine IVF/ICSI treatment, including long-term protocol, short-term protocol, antagonist protocol, and mini stimulus protocol. Those conceived by sperm donation cycles, oocyte donation cycles, intrauterine insemination, or pre-implantation genetic diagnosis cycles were excluded.

Detailed information on maternal and paternal characteristics, ART treatment procedure was obtained from the electronic medical records of reproductive centers. The pregnancy outcomes such as number and sex of offspring were obtained from the follow-up database. Maternal and paternal age was grouped by the means. Body mass index (BMI) was divided into three groups (BMI < l8.5 kg/m^2^, 18.5 kg/m^2^ ≤ BMI < 25 kg/m^2^, BMI ≥ 25 kg/m^2^) according to the World Health Organization criteria. Reproductive history includes infertility type (primary and secondary infertility) and cause of infertility (male factor, female factor, both male and female factor). ART cycle specific parameters, such as ovulation inducing treatments (long-term protocol or other protocols), insemination method (IVF or ICSI), stage of embryos transferred (cleavage stage embryos or blastocyst), and type of embryos (fresh or thawed) were also included in analysis. Ovulation-inducing treatments was divided into two categories of “Long protocol and “other protocol”. “Standard long protocol” is the most conventional treatment and “other protocol” including short protocol and ultra-long protol with different courses, antagonists protocol add GnRH antagonists and micro-stimulation protocol use clomiphene. The infertility factors were clearly diagnosed by clinicians and divided it into three categories including “Female factors”, “Male factors” and “Mixed (both male and female factors)”. “Female factors” include tubal factors, endometriosis, uterine disorders, diminished ovarian reserve, and PCOS. “Male factors” mainly include oligospermia and asthenospermia. The choice of either IVF or ICSI procedure was determined by the patients’ diagnosis. All these factors were included in the final analysis. Pregnancy outcomes were grouped into the type of birth (singletons or twins) and sex at birth. Live birth was defined as any birth event in which at least one baby is born alive. In our analysis SSR (secondary sex ratio) was defined as the percentage of male babies in all live birth.

### Statistical analysis

Pearson χ^2^ test was used to compare the distribution of categorical variables in different subgroups. Unconditional logistic regression was used to estimate the odds ratio (OR), 95% confidence interval and *P* value for the association between selected characteristics and sex ratio. The models were adjusted for some individual factors including maternal age, paternal age and cause of infertility to provide adjusted odds ratio. In our analysis, potential confounders were regarded as covariates that were known associated factors for the birth outcomes and whose distribution between the singleton male and female groups were unequal at a chi-square value of *P* < 0.10. Cause of infertility is an important related factor for assisted reproductive technology treatment and other similar researches about ART birth outcomes also adjusted it refs 15, 17, 20. *P* value < 0.05 was considered to be significant. All analysis was performed with the Statistical Package for the Social Science software (SPSS version 21.0).

## Electronic supplementary material

Supplementary table 1

## References

[1] Pergament E, Toydemir PB, Fiddler M (2002). Sex ratio: a biological perspective of ‘Sex and the City’. Reproductive biomedicine online.

[2] Jongbloet PH (2005). Fetal sex and very preterm birth. Am J Obstet Gynecol.

[3] Orzack SH (2015). The human sex ratio from conception to birth. Proc Natl Acad Sci USA.

[4] Rueness J, Vatten L, Eskild A (2012). The human sex ratio: effects of maternal age. Hum Reprod.

[5] Suzuki K, Yamagata Z, Kawado M, Hashimoto S (2016). Effects of the Great East Japan Earthquake on Secondary Sex Ratio and Perinatal Outcomes. J Epidemiol.

[6] Macmahon B, Pugh TF (1954). Sex ratio of white births in the United States during the Second World War. Am J Hum Genet.

[7] Zadzinska E, Rosset I, Mikulec A, Domanski C, Pawlowski B (2011). Impact of economic conditions on the secondary sex ratio in a post-communist economy. Homo.

[8] Terrell ML, Hartnett KP, Marcus M (2011). Can environmental or occupational hazards alter the sex ratio at birth? A systematic review. Emerg Health Threats J.

[9] Hamoudi A, Nobles J (2014). Do daughters really cause divorce? Stress, pregnancy, and family composition. Demography.

[10] Sunderam S (2015). Assisted Reproductive Technology Surveillance - United States, 2013. Morbidity and mortality weekly report. Surveillance summaries (Washington, D.C.: 2002).

[11] Kissin DM, Jamieson DJ, Barfield WD (2014). Monitoring health outcomes of assisted reproductive technology. The New England journal of medicine.

[12] Griffin DK (1994). Clinical experience with preimplantation diagnosis of sex by dual fluorescent *in situ* hybridization. J Assist Reprod Genet.

[13] Viloria T (2005). Smoking habits of parents and male: female ratio in spermatozoa and preimplantation embryos. Hum Reprod.

[14] Alfarawati S (2011). The relationship between blastocyst morphology, chromosomal abnormality, and embryo gender. Fertil Steril.

[15] Luke B (2009). The sex ratio of singleton offspring in assisted-conception pregnancies. Fertil Steril.

[16] Dean JH, Chapman MG, Sullivan EA (2010). The effect on human sex ratio at birth by assisted reproductive technology (ART) procedures–an assessment of babies born following single embryo transfers, Australia and New Zealand, 2002-2006. BJOG.

[17] Zhu, J. *et al*. Effect of male body mass index on live-birth sex ratio of singletons after assisted reproduction technology. *Fertil Steril* **104**, 1406–1410 e1401–1402, doi:10.1016/j.fertnstert.2015.08.017 (2015). 10.1016/j.fertnstert.2015.08.017 26361206

[18] Alikani M, Cekleniak NA, Walters E, Cohen J (2003). Monozygotic twinning following assisted conception: an analysis of 81 consecutive cases. Hum Reprod.

[19] Chang HJ, Lee JR, Jee BC, Suh CS, Kim SH (2009). Impact of blastocyst transfer on offspring sex ratio and the monozygotic twinning rate: a systematic review and meta-analysis. Fertil Steril.

[20] Fernando D, Halliday JL, Breheny S, Healy DL (2012). Outcomes of singleton births after blastocyst versus nonblastocyst transfer in assisted reproductive technology. Fertil Steril.

[21] Pergament E, Fiddler M, Cho N, Johnson D, Holmgren WJ (1994). Sexual differentiation and preimplantation cell growth. Hum Reprod.

[22] Dumoulin JC (2005). Growth rate of human preimplantation embryos is sex dependent after ICSI but not after IVF. Hum Reprod.

[23] Tarin JJ, Garcia-Perez MA, Hermenegildo C, Cano A (2014). Changes in sex ratio from fertilization to birth in assisted-reproductive-treatment cycles. Reprod Biol Endocrinol.

[24] Shapiro BS, Daneshmand ST, Restrepo H, Garner FC (2012). Serum HCG measured in the peri-implantation period predicts IVF cycle outcomes. Reproductive biomedicine online.

[25] Poikkeus P, Hiilesmaa V, Tiitinen A (2002). Serum HCG 12 days after embryo transfer in predicting pregnancy outcome. Hum Reprod.

[26] Bu Z (2014). Live birth sex ratio after *in vitro* fertilization and embryo transfer in China–an analysis of 121,247 babies from 18 centers. PLoS One.

[27] Setti AS, Figueira RC, Braga DP, Iaconelli A, Borges E (2012). Gender incidence of intracytoplasmic morphologically selected sperm injection-derived embryos: a prospective randomized study. Reproductive biomedicine online.

[28] Xu XK, Wang YA, Li Z, Lui K, Sullivan EA (2014). Risk factors associated with preterm birth among singletons following assisted reproductive technology in Australia 2007-2009–a population-based retrospective study. BMC Pregnancy Childbirth.

[29] Di Renzo GC, Rosati A, Sarti RD, Cruciani L, Cutuli AM (2007). Does fetal sex affect pregnancy outcome?. Gend Med.

[30] Frattarelli JL, Miller KA, Miller BT, Elkind-Hirsch K, Scott RT (2008). Male age negatively impacts embryo development and reproductive outcome in donor oocyte assisted reproductive technology cycles. Fertil Steril.

[31] Vitthala S, Gelbaya TA, Brison DR, Fitzgerald CT, Nardo LG (2009). The risk of monozygotic twins after assisted reproductive technology: a systematic review and meta-analysis. Hum Reprod Update.

