# Supplementary material for: The sex ratio of singleton and twin delivery offspring in assisted reproductive technology in China
Source: Sci Rep. 2017 Aug 24;7:7754. doi: 10.1038/s41598-017-06152-9 (PMC5570918; doi:10.1038/s41598-017-06152-9)
Supplement: Supplementary file 1 — Supplementary table 1 [file 41598_2017_6152_MOESM1_ESM.doc]

**The sex ratio of singleton and twin delivery offspring in assisted reproductive technology in China**

Mengxi Chen, M.D.,^a,b†^ Jiangbo Du, Ph.D.,^a,b†^ Jing Zhao, B.S.,^a,c†^ Hong Lv, M.D.,^a,b^ Yifeng Wang, M.D.,^a,b^ XiaoJiao Chen, M.D.,^a,c^ Junqiang Zhang, M.D., P.H.D.,^a,c^ Lingmin Hu, P.H.D.,^a,d^ Guangfu Jin, M.D., P.H.D.,^a,b^ Hongbing Shen, M.D., P.H.D.,^a,b^ Zhibin Hu, M.D., P.H.D.,^a,b^ Fang Xiong, M.D.,^a,e*^ Li Chen, M.D.,^a,d*^ Xiufeng Ling, M.D., P.H.D.,^a,c*^

^a^ State Key Laboratory of Reproductive Medicine, Nanjing Medical University, Nanjing 211166, China.

^b^ Department of Epidemiology, School of Public Health, Nanjing Medical University, Nanjing 211166, China.

^c^ Department of Reproduction, the Affiliated Nanjing Maternity and Child Health Hospital of Nanjing Medical University, Nanjing 210004, China

^d^ Department of Reproduction, the Affiliated Changzhou Maternity and Child Health Hospital of Nanjing Medical University, Changzhou 213003, China

^e^ Department of Reproduction, the Affiliated Wuxi Maternity and Child Health Hospital of Nanjing Medical University, Wuxi 214002, China

^*^Correspondence to: Xiufeng Ling, Department of Reproduction, the Affiliated Nanjing Maternity and Child Health Hospital of Nanjing Medical University, Nanjing 210004, China, Tel +86-25-5222-6102, E-mail:lingxiufeng_njfy@163.com.

Li Chen, Department of Reproduction, the Affiliated Changzhou Maternity and Child Health Hospital of Nanjing Medical University, Changzhou 213003, China

, Tel +86-519-8811-0326, E-mail: shaoshan686@163.com;

Fang Xiong, Department of Reproduction, the Affiliated Wuxi Maternity and Child Health Hospital of Nanjing Medical University, Wuxi 214002, China

, Tel +86-510-8272-5161, E-mail: xiongfang58@163.com;

^†^ C.M., D.J., Z.J. contributed equally to this work.

| Selected characteristics | Total | Proportion (%) |
| --- | --- | --- |
| All | 5832 |  |
| Infertility type |  |  |
| Primary | 3015 | 53.8 |
| Secondary | 2591 | 46.2 |
| Cause of infertility |  |  |
| Mixed (Both male and female factors) | 1186 | 27.9 |
| Male | 486 | 11.4 |
| Female | 2574 | 60.6 |
| Ovulation-inducing treatments |  |  |
| Long-term protocol | 3351 | 66.1 |
| short protocol | 1401 | 27.7 |
| ultra-long protocol | 111 | 2.2 |
| micro-stimulation protocol | 142 | 2.8 |
| antagonists protocol | 61 | 1.2 |
| Types of embryos transferred |  |  |
| Fresh | 2743 | 47.0 |
| Thawed | 3089 | 53.0 |
| Fertilization methods |  |  |
| IVF | 4210 | 82.9 |
| ICSI | 869 | 17.1 |
| Stage of embryo transferred |  |  |
| Cleavage-stage embryo | 4435 | 78.8 |
| Blastocyst | 1196 | 21.2 |

**Supplementary table 1** The proportion of infertility and therapeutic factors of the total treatment circles.
